# Telemedicine-based inspiratory muscle training and walking promotion with lung cancer survivors following curative intent therapy: a parallel-group pilot randomized trial

**DOI:** 10.1007/s00520-023-07999-7

**Published:** 2023-09-01

**Authors:** Duc M. Ha, Angela Comer, Blythe Dollar, Ruth Bedoy, Morgan Ford, Wendolyn S. Gozansky, Chan Zeng, Joanna J. Arch, Heather J. Leach, Atul Malhotra, Allan V. Prochazka, Robert L. Keith, Rebecca S. Boxer

**Affiliations:** 1 Institute for Health Research, Kaiser Permanente Colorado2550 S Parker Rd Suite 200, Aurora, CO 80014 USA; 2 Section of Pulmonary and Critical Care, Medical & Research Service, Rocky Mountain Regional Veterans Affairs Medical Center, Aurora, CO USA; 3 Department of Medicine, University of Colorado Anschutz Medical Campus, Aurora, CO USA; 4 Department of Psychology and Neuroscience, University of Colorado Boulder, Boulder, CO USA; 5 Cancer Prevention and Control, University of Colorado Cancer Center, Aurora, CO USA; 6 Department of Health and Exercise Science, Colorado State University, Fort Collins, CO USA; 7 Division of Pulmonary, Critical Care, Sleep Medicine and Physiology, University of California San Diego, San Diego, CA USA; 8 Division of Geriatrics, Hospice and Palliative Care Medicine, University of California Davis, Sacramento, CA USA

**Keywords:** Rehabilitation, Telerehabilitation, Telemedicine, Dyspnea, Exercise, Survivorship, Patient-centered outcomes

## Abstract

**Purpose:**

Following curative-intent therapy of lung cancer, many survivors experience dyspnea and physical inactivity. We investigated the feasibility, acceptability, safety, and potential efficacy of inspiratory muscle training (IMT) and walking promotion to disrupt a postulated “dyspnea-inactivity” spiral.

**Methods:**

Between January and December 2022, we recruited lung cancer survivors from Kaiser Permanente Colorado who completed curative-intent therapy within 1–6 months into a phase-IIb, parallel-group, pilot randomized trial (1:1 allocation). The 12-week intervention, delivered via telemedicine, consisted of exercise training (IMT + walking), education, and behavior change support. Control participants received educational materials on general exercise. We determined feasibility a priori: enrollment of ≥ 20% eligible patients, ≥ 75% retention, study measure completion, and adherence. We assessed acceptability using the Telemedicine-Satisfaction-and-Usefulness-Questionnaire and safety events that included emergency department visits or hospitalizations. Patient-centered outcome measures (PCOMs) included dyspnea (University-of-California-San-Diego-Shortness-of-Breath-Questionnaire), physical activity (activPAL™ steps/day), functional exercise capacity (mobile-based-six-minute-walk-test), and health-related quality of life (HRQL, St.-George’s-Respiratory-Questionnaire). We used linear mixed-effects models to assess potential efficacy.

**Results:**

We screened 751 patients, identified 124 eligible, and consented 31 (25%) participants. Among 28 participants randomized (14/group), 22 (11/group) completed the study (79% retention). Intervention participants returned > 90% of self-reported activity logs, completed > 90% of PCOMs, and attended > 90% of tele-visits; 75% of participants performed IMT at the recommended dose. Participants had high satisfaction with tele-visits and found the intervention useful. There was no statistically significant difference in safety events between groups. Compared to control participants from baseline to follow-up, intervention participants had statistically significant and clinically meaningful improved HRQL (SGRQ total, symptom, and impact scores) (standardized effect size: -1.03 to -1.30).

**Conclusions:**

Among lung cancer survivors following curative-intent therapy, telemedicine-based IMT + walking was feasible, acceptable, safe, and had potential to disrupt the “dyspnea-inactivity” spiral. Future efficacy/effectiveness trials are warranted and should incorporate IMT and walking promotion to improve HRQL.

**Trial Registration:** ClinicalTrials.gov NCT05059132.

**Supplementary information:**

The online version contains supplementary material available at 10.1007/s00520-023-07999-7.

## Introduction

Dyspnea is an important patient-centered outcome impacting health-related quality of life (HRQL) in lung cancer [1]. High dyspnea burden may reduce functional exercise capacity [2] and survival [3]. Compared to pretreatment, dyspnea worsens among lung cancer survivors following curative intent therapy [4–7] and can persist for years posttreatment, regardless of treatment modality [8–10]. Pathophysiologically, dyspnea can occur due to resected or damaged lung tissue, lost or damaged nerve fibers [11], and activation or increased stimulation of peripheral sensors [12]. Along with alterations in the chest wall, respiratory muscle, and airway [13], these changes can culminate in neuromechanical dissociation and increase central ‘corollary discharge’ [13]. In fact, clinically significant dyspnea exists among up to 70–80% of lung cancers survivors within six months following curative intent therapy [5, 14] and 60% among those ≥ 1 year/s posttreatment [15]. As such, dyspnea is a modifiable factor that could be targeted to improve HRQL following curative intent therapy [16].

A psychological consequence of dyspnea is fear, panic, and anxiety, particularly with exertion [17]. A behavioral consequence of dyspnea [17] is avoidance of physical activity and exercise [18], with physical inactivity associated with poor HRQL [19] and worse survival in early stage lung cancer [20]. The American College of Chest Physicians identified a need for strategies to improve HRQL following curative intent therapy of lung cancer [21]. Accordingly, we proposed a conceptual model of a vicious cycle of “dyspnea-inactivity” downward health spiral that needs to be promptly disrupted for this unique [2] and growing population of cancer survivors [22].

Inspiratory muscle training (IMT) is a resistance-based exercise training regimen to improve strength and endurance of respiratory muscles. IMT alleviates dyspnea for patients with chronic obstructive pulmonary disease (COPD) [23], can be performed in patients’ homes, and is a promising strategy to meet lung cancer survivors’ needs for remotely-delivered rehabilitation [24]. In addition, walking is the preferred physical activity modality among lung cancer survivors [25]. Therefore, IMT and walking promotion may disrupt the vicious cycle of”dyspnea-inactivity.” In this project, we conducted a pilot randomized trial of a telemedicine-based rehabilitation strategy consisting of IMT + walking with lung cancer survivors following curative intent therapy. We hypothesized that IMT + walking is feasible, acceptable, safe, and compared to education only, could improve dyspnea control, physical activity, functional exercise capacity, and HRQL.

## Methods

### Trial design & study overview

We registered this protocol (NCT05059132) and designated physical activity as a primary outcome. We applied the ORBIT model for developing behavioral treatments [26] and conducted a phase IIb, parallel group, pilot randomized trial (1:1 allocation). This study received approval and waiver of signed informed consent from the Kaiser Permanente Colorado Institutional Review Board (#1,717,517–12). Participants’ verbal informed consent was obtained by telephone following a discussion of the study, contained all the required elements of informed consent, and documented in REDCap – a secure electronic data management system [27]; a copy of the informed consent form was sent to participants thereafter. To report findings, we followed the Consolidated Standards of Reporting Trials, pilot extension [28].

### Recruitment & participants

Between January and December 2022, we recruited patients from Kaiser Permanente Colorado (KPCO), an integrated healthcare system that provides health insurance and clinical services to > 500,000 individuals in the metropolitan Denver and surrounding Colorado communities. We used a multi-modal recruitment approach: 1) identification of patients with receipt of curative intent therapy; 2) new referrals to the pulmonology, surgery, or radiation oncology departments for newly-diagnosed or suspected lung cancer; and 3) patients presented at a weekly lung cancer/nodule conference. To facilitate recruitment, we developed an algorithm of codes and local chemoradiation protocols, incorporating relevant time periods and exclusionary conditions (Online Resource 1). We reviewed the records of patients identified and sent recruitment letters to those deemed potentially eligible. We allowed two weeks for patients to decline recruitment and reached out to those who did not decline.

We included adult stage I-IIIA lung cancer survivors who completed the primary mode of curative intent therapy (i.e., surgical resection, definitive radiation, or concurrent chemoradiation) in the prior 1–6 months. We excluded patients with: 1) recent major cardiovascular events or acute asthma exacerbation; 2) spontaneous pneumothorax within 12 months; 3) neurologic or movement disorders; 4) dementia; 5) estimated < 6-months life expectancy or in hospice care; 6) no internet access; 7) inactive KPCO membership; 8) any preferred language other than English; or 9) unwilling to wear activity trackers. We included patients not willing to participate in telemedicine, as we would allow in-person visits, if needed. We obtained demographic, physiologic, and clinical characteristics from the electronic medical records.

### Randomization

Participants who completed baseline patient-centered outcome measures (PCOMs) were randomized in permuted blocks of four, stratified by receipt of surgical or non-surgical treatment, and allocated 1:1 to the IMT + walking (intervention) or education only (control) groups. A computer-generated allocation sequence was uploaded onto REDCap [27]. The study investigators, but not participants, interventionist, or outcome assessor, were blinded to group allocation.

### IMT + walking (Intervention)

Participants in the IMT + walking group received an intervention designed with essential components of pulmonary rehabilitation (i.e., exercise training, education, behavioral support) [29], delivered in six tele-visits over 12 weeks. Exercise training consisted of adapted IMT [30] + walking [31], guided by exercise recommendations to improve HRQL for cancer survivors [i.e., moderate-vigorous intensity physical activity (MVPA) ≥ 60–90 min/week for 12 weeks] [32]. Education focused on the potential of IMT + walking to improve dyspnea control, function, HRQL, and incorporated patient educational materials on dyspnea [33], IMT, and physical activity [34]. Behavioral support was informed by the 2018 Physical Activity Guidelines Advisory Committee (PAGAC) Scientific Report from the US Department of Health and Human Services, which concluded that “strong evidence demonstrates that behavior change theory and techniques are effective for increasing physical activity levels in general adult populations” [35]. We adapted Bandura’s Social Cognitive Theory (SCT) [36], a framework identified to be effective in this Scientific Report [35] with supporting evidence among cancer survivors [37]. The SCT postulates that knowledge of health risks and benefits initiates the process of possible behavior change, with behavior influenced by 1) perceived self-efficacy, 2) facilitators and impediments, 3) outcome expectations, and 4) goals. Guided by the SCT, behavioral support incorporated behavior change techniques shown to be effective in promoting habitual exercise among general adults [35] and cancer survivors [38] – i.e., providing information on the expected benefits of IMT + walking, clear instructions on how to perform IMT to promote self-efficacy, setting achievable activity goals, graded tasks, self-monitoring, identifying barriers and facilitators, problem-solving, and feedback. We provided descriptions of how the SCT and behavior change techniques were applied in the intervention in Table 1 and Online Resource 2a-b. We did not combine behavior change theories and avoided behavior change techniques identified by the PAGAC to be likely ineffective (e.g., general encouragement) [35].

**Table 1:** Targeted Intervention^*^ to Disrupt the Vicious Cycle of “Dyspnea-Inactivity”

Exercise Training (IMT + Walking)
• RS introduces IMT and explains how IMT can alleviate dyspnea • RS demonstrates IMT technique via video conferencing (*information on how)* • RS asks participants to perform IMT for 5–10 min under video observation and provides supportive feedback (prompt practice; self-efficacy^**^) • RS instructs participants to perform unsupervised IMT at Borg rating of perceived exertion “somewhat-hard to hard (i.e., 4–6 on 0–10 scale), 10–15 min twice daily, ≥ 5 days/week (goal-setting^**^), with increased resistance levels as tolerated (*graded tasks*) **+ ** • RS explains the importance of regular walking to promote health • RS reviews Fitbit step count with participant and discuss walking bouts (*prompt review of behavior*) • RS advises 5–10% increase from baseline in average daily step count (goal-setting^**^) • RS advises engagement in ≥ 4 walking bouts/week, or 5–10 min increases from baseline walking durations, to meet step count goal (*graded tasks*)
Education
• RS explains the role of IMT + walking to alleviate dyspnea, improve function, and HRQL following lung cancer treatment (information about benefits; outcome expectations^**^)
Behavior Change Support^**^
• RS and participant collaboratively discuss potential challenges to IMT + walking (barriers/facilitators^**^) • RS provides prescription for IMT + walking, discuss, and collaboratively formulate activity goals in subsequent weeks (goal-setting^**^) • RS explains and encourages the use of Fitbit device and activity logs to facilitate monitoring of IMT + walking goals (*self-monitoring*) • RS and participant collaboratively identify strategies to minimize barriers and promote facilitators towards IMT + walking (*problem-solving*) • RS encourages participant to engage in regular IMT + walking and congratulates participants on achieving goals (*feedback/encouragement*)

^*^Guided by essential components of pulmonary rehabilitation (i.e., exercise training, education, behavioral support) [29] and *the Social Cognitive Theory (SCT) *[36]

^**^Constructs of the SCT (i.e., self-efficacy, outcome expectations, barriers/facilitators, goals) [36]

Italicized text represents behavioral change techniques and/or SCT constructs [38]

HRQL = health-related quality of life; IMT = inspiratory muscle training; RS = research specialist (interventionist); SCT = Social Cognitive Theory

Tele-visits were scheduled at weeks 1, 3, 5, 7, 9, and 12 following randomization and lasted 30–60 min/visit. All six visits involved live interventionist interaction via telemedicine, with IMT and Fitbit devices sent via parcel services. The initial tele-visit focused on introduction to IMT + walking and expected benefits, instructions on how to perform IMT, setting activity goals for the coming weeks, and the use of activity logs and activity trackers for self-monitoring. The subsequent tele-visits focused on behavioral support, with collaborative review of activity goals achieved, identification of barriers and facilitators, problem-solving, and feedback.

#### Inspiratory muscle training

IMT was performed using Threshold IMT™ devices (Philips Healthcare), with instructions provided via video-visits to allow observation, feedback, and ensure IMT proficiency. Participants were instructed to adjust IMT resistance to a perceived rating of exertion of “somewhat-hard to hard” (4–6 on the 0–10 modified Borg scale), perform unsupervised IMT 10–15 min twice/day (or ≥ 20 min/daily) for ≥ 5 days/week (i.e., IMT ≥ 100 min/week), with progression to higher resistance as tolerated. Once participants have demonstrated proficiency with IMT in video-visit/s, telephone visits were allowed if participants encountered significant technical difficulties (e.g., loss of internet signal).

#### Walking

Walking promotion was facilitated by patient-facing activity trackers (Fitbit Inspire 2™) for activity goal-setting, self-monitoring, and feedback. To obtain baseline step count, we sent Fitbits to participants, assisted them with device/account set-up, and instructed one-week wear prior to the initial tele-visit. The activity goal was 5–10% increases from baseline and previous weeks’ step counts. To achieve activity goals, participants were encouraged to go on ≥ 4 walks/week or increase walk durations by 5–10 min. To facilitate self-monitoring and adherence, participants were asked to access Fitbit data and self-record step counts and IMT sessions on activity logs, completed weekly, returned, and discussed at each tele-visit.

#### *Interventionist*

To deliver the intervention, a research specialist completed coursework in motivational interviewing [39] and received training in IMT with support from respiratory therapists. Microsoft Teams™ was used for video-conferencing, with telephone used as needed if participants had demonstrated adequate IMT proficiency. To ensure fidelity, the interventionist used a checklist of components (Online Resource 2a-b), with 10% of completed checklists reviewed to identify and address any challenges to delivery.

### Education only (Control)

Control group participants received written educational materials – on physical activity in lung cancer [34]; sitting less/moving more [40]; and being physically active [41] – sent via email or post at weeks 1, 4, and 8. There were no additional monitoring or contact with study personnel, except at weeks 6 and 12 for study outcome assessments. These participants received IMT and Fitbit devices at study end.

### Outcomes

#### Feasibility, Acceptability, and Safety

We determined feasibility a priori, guided by frameworks for pilot randomized rehabilitation trials [42, 43]: enrollment (i.e., ≥ 20% of eligible patients), randomization (i.e., participant willingness to be randomized), participant adherence (i.e., attendance of ≥ 75% of tele-visits; performance of ≥ 75% unsupervised IMT + walking), interventionist fidelity, measurement processes, and retention (i.e., ≥ 75%). We chose a higher-than-recommended completion threshold (70% for quality pulmonary rehabilitation) [44] due to the mostly unsupervised nature of IMT + walking, as the effects of unsupervised exercise on HRQL have been shown to be smaller compared to supervised exercise [45].

Acceptability was measured by the Telemedicine Satisfaction and Usefulness Questionnaire (1–5 Likert scale responses; scores ≥ 4 indicate acceptable satisfaction and usefulness) [46] and an exit survey on participants’ experience with the intervention. To assess safety, we identified episodes of emergency department visits or hospitalizations for participants in both groups, and any self-reported symptom/signs associated with IMT + walking.

#### Patient-centered outcome measures

All PCOMs were measured at time points 0 (baseline), week 6 (mid-intervention), and week 12 (end-of-intervention): dyspnea [UCSD Shortness of Breath Questionnaire (SOBQ), 0–120 point scale, higher scores indicate higher dyspnea]; anxiety [Generalized Anxiety Disorder 7-item (GAD-7), 0–21 point scale, higher scores indicate higher anxiety], sleep quality [Pittsburgh Sleep Quality Index (PSQI), 0–21 point scale, higher scores indicate worse sleep quality]; and self-reported physical activity [International Physical Activity Questionnaire – Short Form, minutes/week of walking and MVPA].

Physical activity was measured by the activPAL (4 micro, PAL™ Technologies), a valid and accurate wearable monitor to measure physical activity (stepping, step counts, step speed, postural transitions) and sedentary behavior (sitting/lying) [47] that has been used among survivors of cancer [48, 49], including lung [50]. We used a 7-day continuous wear protocol [47] and default settings/algorithms to define valid data – i.e., days with non-wear ≤ 4 h, from midnight to the next midnight, supplemented by self-reported wear/sleep logs. activPAL measures were steps/day, sedentary behavior (SB) (minutes/day), light intensity physical activity (LPA) (minutes/day with cadence < 100 steps/min), and MVPA (minutes/day with cadence ≥ 100 steps/min).

Functional exercise capacity was measured by the mobile-based six-minute walk test (m6MWT). To conduct the m6MWT, we modified the American Thoracic Society recommendations for in-person performance [51], completed the test remotely, and with live interaction (via telephone) with study personnel for monitoring. We asked participants to identify an acceptable path (flat, without traffic, and approximately 1/3^rd^ to 1/4^th^ the length of a typical city block – approximately 30 m) to walk back and forth. Recording of the m6MWT distance was enabled by the 6WT application (Webgearing AG). The m6MWT distance has been shown to be reliable, reproducible, and concordant (or accurate) with in-lab measures in adults [52] and patients with cardiopulmonary disease [53, 54]. We used reference equations from healthy adults [55] to calculate lower limits of normal for interpretation. Please also see Online Resource 3 for more details.

HRQL was measured by the 50-item St. George’s Respiratory Questionnaire (SGRQ), with 1) symptoms (frequency, severity); 2) activities (causing or limited by dyspnea); 3) impact (on social functioning and psychological disturbance); and total scores ranging 0–100 points; lower scores indicate improved HRQL. Online Resource 4 provides more PCOM details, including cut-off levels and minimal clinically important difference (MCID) thresholds.

### Sample Size

We aimed 30–40 participants enrolled (15–20/group) based on estimates for a continuous outcome variable in a pilot trial [56] to minimize overall (pilot and efficacy) trial sample size, and detect an estimated standardized medium (0.3–0.7) effect size in an efficacy trial [56].

We used the MCID 350–1,100 steps/day (derived in COPD) [57]. We assumed a baseline activPAL 3,500 ± 2,100 steps/day [50], 40 participants randomized (20/group), 20% dropout, and with 80% power (two-sided alpha = 0.05), estimated that we would be able to detect a mean difference in response of 1,910 steps/day between groups, a very-large standardized effect size (SES 0.9) and approximately 2–5 times the MCID.

### Statistical Analyses

Descriptive statistics summarized participant characteristics and two-sample t-test and Chi-square or Fisher’s exact tests compared differences between groups at baseline. Linear mixed-effects models analyzed change in PCOMs. We followed recommendations against hypothesis testing in pilot trials [28] and used unadjusted models in primary analyses, with each PCOM modeled as function of group assignment, visit week, and interaction of group by visit week. To assess potential treatment effects, changes in PCOMs from baseline were estimated and compared between groups. We used all available data. Linear mixed-effects models assumed random missing data.

To assess the validity of the “dyspnea-inactivity” spiral, we used adjusted models in secondary analyses, including participant’s age and comorbidities [58]. We did not adjust for multiple comparisons as our trial was not designed to determine efficacy/effectiveness. We used SAS/STAT analytic software (V.9.4 SAS Institute Inc).

## Results

### Screening and enrollment

**Fig. 1:**
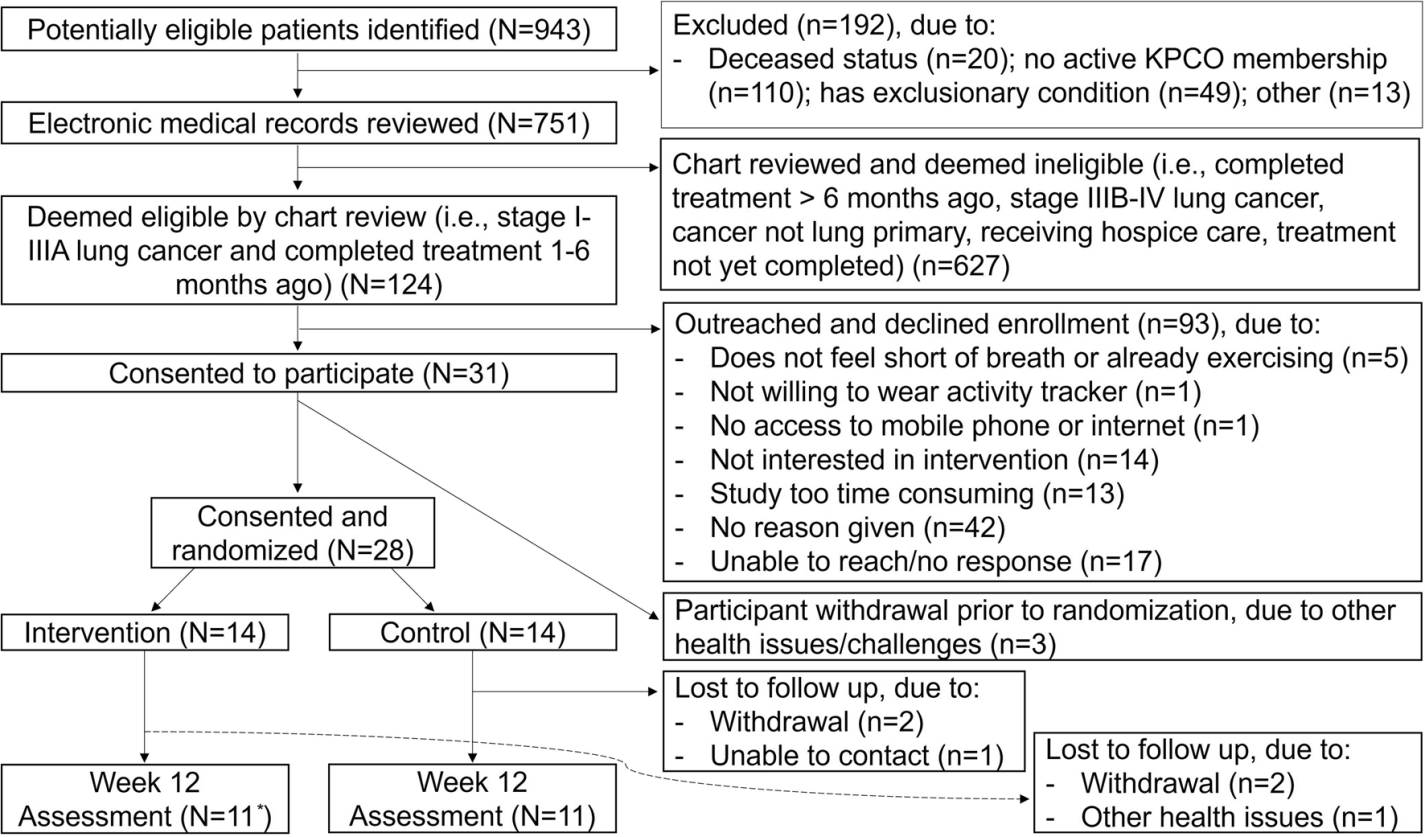
Consort Diagram. ^*^Including one participant who completed but did not return the activPAL device. KPCO = Kaiser Permanente Colorado

We screened 751 patients, identified 124 eligible stage I-IIIA lung cancer survivors, and consented 31 participants (25% enrollment); the most common reported reasons for non-participation were high time commitment and low interest. Three participants withdrew prior to randomization (Fig. 1). Among 28 participants randomized (14/group), the median age was 70 years; approximately 50% were women, 30% had comorbid COPD, 80% stage IA, and 60% received only surgical treatment (Table 2).

**Table 2:** Baseline Participant Characteristics

Participant Characteristics	Control (*n* = 14)	Intervention (*n* = 14)	*p* value
Age, years, mean ± SD	70.3 ± 7.4	68.3 ± 6.4	0.65
Women, %	50	57	0.71
Race/ethnicity, %			
White, non-Hispanic	100	79	0.19^*^
Multiple	0	7	
Unknown	0	14	
Married marital status, %	79	57	0.32^*^
BMI, kg/m^2^, mean ± SD	26.7 ± 4.2	28.2 ± 3.9	0.46
Smoking status, %			
Current	7	7	0.18^*^
Former	86	57	
Never	7	36	
Pack years, mean ± SD	40.1 ± 18.4	29.2 ± 13.7	0.22
Comorbidity score^*a*^, mean ± SD	3.7 ± 2.8	3.1 ± 1.4	0.74
Spirometry, mean ± SD			
FEV_1_, % predicted	86.1 ± 25.4	77.3 ± 20.1	0.19
FVC, % predicted	95.0 ± 12.6	78.7 ± 14.5	0.11
FEV_1_/FVC ratio	70.1 ± 9.0	77.4 ± 7.1	0.19
Spirometric impairment, %			
Obstructive (FEV_1_/FVC < 70%)	33	25	0.71^*^
Lung cancer characteristics			
Histologic subtype, %			
Adenocarcinoma	57	79	0.32^*^
Squamous cell	14	14	
NSCLC-NOS	7	7	
Presumed lung cancer	21	0	
Collaborative stage, %			
IA	86	79	0.59^*^
IB	0	7	
IIB	14	14	
Primary curative intent therapy, %			
Surgery only	57	64	0.98^*^
Surgery + adjuvant chemotherapy	7	7	
SBRT only	29	21	
Conventional radiation only	7	7	
Months since treatment completion			
Mean ± SD	3.3 ± 1.9	2.9 ± 1.5	0.53
Median (Min—Max)	3.0 (1.0—6.0)	2.5 (1.0—5.0)	
PCOMs			
SOBQ, points			
Mean ± SD	32.0 ± 20.3	34.5 ± 18.3	0.55
activPAL, mean ± SD			
PA, steps/day	6,722 ± 3292	5,784 ± 2000	0.41
SB-1^*b*^, min/day	625.1 ± 217.3	566.9 ± 139.3	0.34
SB-2^*c*^, min/day	638.5 ± 214.0	648.7 ± 106.1	0.93
LPA, min/day	172.3 ± 114.4	182.3 ± 84.0	0.41
MVPA, min/day	12.2 ± 14.0	11.3 ± 8.1	0.58
GAD-7, points			
Mean ± SD	5.86 ± 6.36	3.00 ± 2.45	0.61
m6MWT			
Distance, meters, mean ± SD	403.8 ± 166.0	474.5 ± 190.4	0.34
BDS, points, mean ± SD			
Pre-m6MWT	1.58 ± 1.98	0.73 ± 0.79	0.36
Post-m6MWT	5.83 ± 2.89	5.36 ± 2.80	0.80
Change	4.25 ± 2.93	4.64 ± 2.84	0.71
PSQI, points			
Mean ± SD	6.79 ± 2.97	7.79 ± 3.09	0.38
SGRQ, points, mean ± SD			
Total	33.7 ± 15.9	41.2 ± 14.4	0.10
Symptoms	46.2 ± 21.4	56.3 ± 19.2	0.23
Activities	48.7 ± 23.0	55.2 ± 22.8	0.44
Impact	20.7 ± 15.5	27.3 ± 16.4	0.24
IPAQ-SF, mean ± SD			
SB/Sitting, min/day	426.4 ± 189.8	309.8 ± 217.2	0.12
Walking (LPA), min/wk	284.3 ± 521.4	434.3 ± 550.0	0.09
MVPA, min/wk	696.8 ± 1226	367.5 ± 531.9	0.58

^*^p value from exact test

^*a*^*As defined by the Quan-Elixhauser comorbidity index *[58]

^*b*^*Sitting time only*

^*c*^*Siting time (obtained from the activPAL)* + *awake lying time (obtained from self-reported sleep log)*

BDS = Borg Dyspnea Score; BMI = body-mass index; FEV_1_ = forced expiratory volume in 1 s; FVC = forced vital capacity; GAD-7 = Generalized Anxiety Disorder – 7 item; IMT = inspiratory muscle training; IPAQ-SF = International Physical Activity Questionnaire – Short Form; LPA = light intensity physical activity; m6MWT = mobile-based six-minute walk test; MVPA = moderate-vigorous intensity physical activity; NSCLC-NOS = non-small cell lung cancer, not otherwise specified histologic subtype; PA = physical activity; PCOM = patient-centered outcome measure; PSQI = Pittsburgh Sleep Quality Index; SB = sedentary behavior; SBRT = stereotactic body radiotherapy; SD = standard deviation; SGRQ = St. George’s Respiratory Questionnaire; SOBQ = University of California San Diego Shortness of Breath Questionnaire

### Baseline participant characteristics

Ninety-three percent of participants had abnormally-high dyspnea (SOBQ > 9 points), 93% low physical activity (activPAL < 10,000 steps/day), 86% disrupted sleep (PSQI ≥ 5 points), and 89% poor HRQL (SGRQ > 13 points). Most had minimal anxiety (GAD-7 ≤ 4 points, 64%) and adequate functional exercise capacity (m6MWT distance ≥ lower limit of normal, 83%). There were no statistically significant differences in participant characteristics, including PCOMs, between groups at baseline (Table 2).

### Intervention feasibility and acceptability

Among 28 participants randomized, 22 (11/group) completed the study (79% retention); > 95% of the PCOMs were obtained, with approximately 90% of participants completing 100% of PCOMs. The most common missing PCOM was the m6MWT – with eight (12%) (four/group) not completed due to unreliable mobile phone signal, unacceptable walking space, or weather challenges.

Among 11 participants who completed the intervention, > 90% attended ≥ 75% of tele-visits, with 75% of them attending 100% of visits. Approximately 90% of activity logs were returned, with 75% of participants performing IMT at the prescribed dose – i.e., ≥ 100 min/week, and/or walking ≥ 90 min/week (Table 3a). Adherence was sustained (Online Resource 5). The research specialist delivered > 95% of the checklist items without difficulty, with no changes made during the trial. All visits were via telemedicine, mostly with video and approximately 20% with telephone due to technical challenges; there were no in-person visits.

**Table 3:** Feasibility, Acceptability, and Safety Measures

***(a) Feasibility***	
*Participant* *adherence* *to IMT + walking (intervention group* *only,* *n* *= 11)*	Value
Performed IMT ≥ once daily, 5 days/week, %	89
Performed IMT ≥ twice daily, 5 days/week, %	74
Performed IMT ≥ 100 min/week, %	75
Minutes performed IMT per week, mean ± SD	137 ± 50
Completed ≥ 1 walk/week, %	87
Completed ≥ 4 walks/week, %	63
Walked ≥ 90 min/week, %	76
Minutes walked per week, mean ± SD	200 ± 199
*Attendance and completion (intervention group only,* *n* *= 11)*	
Participants completing ≥ 75% of scheduled tele-visits, %	92
Participants completing 100% of scheduled tele-visits, %	75
*Measurement processes*	
Activity logs returned, % (intervention group only, *n* = 11)	92
Participants returning ≥ 75% of activity logs (intervention group only, *n* = 11)	91
PCOMs obtained, % (both groups, *N* = 22)	98
Participants with 100% of PCOMs completed at week 12, % (both groups, *N* = 22)	91
***(b) Acceptability***	
*Telemedicine Satisfaction and Usefulness Questionnaire*^a^ *(n* *= 11)*	
The lack of physical contact during a video visit is not a problem, %	91
My privacy is protected during a video visit, %	82
Talking to the study team during a video visit is as satisfying as talking in person, %	73
Video visits make it easier for contacts, %	91
Video visits save me time, %	91
The software for video visits is easy to use, %	82
I can always trust the technology to work, %	36
In general, I am satisfied with the video visit system, %	91
It was easy to learn to learn the breathing exercise with video visits, %	91
*Exit Survey*^b^ *(n* *= 11)*	
Instructions and prescriptions for breathing exercises were helpful, %	100
Having access to the breathing (IMT) device was helpful, %	91
Instructions and prescriptions for walking and step count were helpful, %	91
Having access to the Fitbit device was helpful, %	91
Tele-coaching visits were helpful, %	100
Educational documents on shortness of breath, exercise, and physical activity and lung cancer were helpful, %	46
Program overall was helpful, %	91
Would you repeat this or another similar program? (“Yes”), %	100
Would you recommend this program to another patient? (“Yes”), %	100
***(c) Safety***	
Participants with ≥ 1 ED visit and/or hospitalization/s, %	27
Intervention group	9
Control group	45
Intervention participants who reported ≥ 1 symptom/s or sign/s potentially related to IMT + walking, %	54
Fall or other serious symptom/sign	0

^*a*^*Agree or strongly agree*

^*b*^*Moderately, very, or extremely helpful*

ED = emergency department; IMT = inspiratory muscle training; PCOM = patient-centered outcome measure; SD = standard deviation

Participants found tele-visits to be acceptable and were satisfied, noting that the software was easy to use and that tele-visits saved time. However, only 73% of participants indicated that tele-/video-visits were as satisfying as in-person visits, with 36% trusting the technology to work. Ninety to 100% of participants found instructions for IMT + walking as moderately-to-extremely helpful and would recommend the study to another or repeat a similar program. The least helpful component was written educational materials (Table 3b).

### Intervention safety

Of 11 intervention participants who completed the program, six (55%) reported ≥ 1 symptom/s potentially related to IMT + walking (i.e., musculoskeletal soreness, fatigue, lightheadedness, headache, coughing, or breathlessness). There were no serious safety events (e.g., falls with walking) attributable to the intervention. The proportion of participants with emergency department visits or hospitalizations was not statistically significantly different, but appeared lower, in the intervention compared to control group (9 vs 46%, respectively, *p* = 0.15) (Table 3c).

### Change in patient-centered outcome measures

The mean estimated changes in PCOMs from unadjusted models are in Table 4. Compared to control participants from baseline to follow up at 6- or 12-weeks, intervention participants had statistically significant (*p* < 0.05) improved activPAL MVPA and reduced activPAL SB at week 6 (SES 1.08 and -0.94, respectively) but not week 12, and improved HRQL (SGRQ total, symptom, and impact subdomains) at weeks 6 and 12 (SES ranged -1.03 to -1.30). The magnitude of the change differences between groups were ≥ 1–4 times the respective MCIDs and not driven by outliers (Online Resource 6). There were no statistically significant differences in the changes in SOBQ dyspnea, activPAL steps/day or LPA, anxiety, functional exercise capacity, sleep difficulties, or self-reported physical activity between groups at 6 or 12 weeks. Results were similar in adjusted compared to unadjusted models, including in the trends, directionality, and magnitude of potential treatment effects on PCOMs.

**Table 4:** Change in Patient-Centered Outcome Measures^*a*^

		Estimated change from baseline, Mean (95% CI)				
Outcome measures	Week	Control (Education only)	Intervention (IMT + walking)	Difference between groups, Mean (95% CI)	SES^*b*^	*p* value for difference between groups
SOBQ, points	6	-1.74 (-8.79, 5.31)	-9.42 (-16.5, -2.37)^**^	-7.68 (-17.6, 2.29)	-0.59	0.13
	12	-1.90 (-9.18, 5.38)	-4.09 (-11.4, 3.19)	-2.20 (-12.5, 8.10)	-0.16	0.67
activPAL						
PA,steps/day	6	229.3 (-1231, 1689)	1,992 (584.3, 3399)^**^	1762 (-266, 3791)	0.66	0.09
	12	372.9 (-1087, 1833)	1,304 (-201, 2808)	930.7 (-1166, 3027)	0.34	0.38
SB-1^*c*^, min/day	6	-9.78 (-55.3, 35.8)	-11.4 (-55.1, 32.2)	-1.65 (-64.7, 61.5)	-0.02	0.96
	12	-27.6 (-73.1, 18.0)	-10.1 (-56.9, 36.7)	17.4 (-47.8, 82.8)	0.20	0.59
SB-2^*d*^, min/day	6	-8.90 (-58.6, 40.8)	-93.6 (-141, -45.9)^***^	-84.7 (-154, -15.8)	-0.94	0.02
	12	-38.6 (-88.3, 11.2)	-64.1 (-115, -13.0)^*^	-25.5 (-96.8, 45.8)	-0.27	0.47
LPA, min/day	6	5.64 (-23.4, 34.7)	16.6 (-11.2, 44.5)	11.0 (-29.3, 51.3)	0.21	0.58
	12	20.7 (-8.41, 49.7)	3.04 (-26.8, 32.9)	-17.6 (-59.3, 24.1)	-0.32	0.40
MVPA, min/day	6	-2.30 (-9.28, 4.69)	11.5 (4.72, 18.2)^***^	13.7 (4.05, 23.4)	1.08	0.01
	12	-2.15 (-9.14, 4.83)	6.04 (-1.16, 13.2)	8.19 (-1.84, 18.2)	0.62	0.11
GAD-7, points	6	-0.47 (-2.09, 1.15)	0.17 (-1.45, 1.78)	0.64 (-1.65, 2.92)	0.21	0.58
	12	-0.19 (-1.86, 1.48)	0.16 (-1.50, 1.83)	0.36 (-2.00, 2.72)	0.12	0.76
m6MWT						
Distance, meters	6	-14.4 (-129, 100.0)	-42.0 (-159, 74.6)	-27.6 (-191, 135.7)	-0.13	0.73
	12	-0.52 (-111, 109.7)	-82.0 (-197, 32.5)	-81.5 (-240, 77.4)	-0.39	0.31
Post-m6MWT BDS, points	6	-1.03 (-2.40, 0.34)	-0.30 (-1.66, 1.07)	0.73 (-1.20, 2.67)	0.29	0.45
	12	-0.80 (-2.05, 0.46)	-0.28 (-1.58, 1.02)	0.52 (-1.29, 2.32)	0.22	0.57
PSQI, points	6	-1.00 (-2.38, 0.38)	0.41 (-0.97, 1.78)	1.41 (-0.54, 3.35)	0.55	0.15
	12	-1.71 (-3.13, -0.29)^*^	-0.04 (-1.46, 1.38)	1.66 (-0.35, 3.67)	0.63	0.10
SGRQ Total, points	6	-0.53 (-6.84, 5.79)	-13.6 (-19.9, -7.30)^***^	-13.1 (-22.0, -4.16)	-1.12	0.01
	12	3.02 (-3.50, 9.54)	-12.7 (-19.2, -6.16)^***^	-15.7 (-24.9, -6.49)	-1.30	0.00
SGRQ symptom, points	6	-3.42 (-12.7, 5.90)	-22.4 (-31.4, -13.3)^***^	-18.9 (-31.9, -5.94)	-1.11	0.01
	12	-2.07 (-11.7, 7.56)	-20.2 (-29.5, -10.8)^***^	-18.1 (-31.5, -4.68)	-1.03	0.01
SGRQ activity, points	6	-1.73 (-10.6, 7.19)	-7.16 (-16.1, 1.75)	-5.44 (-18.0, 7.18)	-0.33	0.39
	12	5.86 (-3.35, 15.1)	-6.84 (-16.1, 2.37)	-12.7 (-25.7, 0.33)	-0.74	0.06
SGRQ impact, points	6	1.00 (-6.82, 8.82)	-14.5 (-22.4, -6.73)^***^	-15.5 (-26.6, -4.50)	-1.07	0.01
	12	2.47 (-5.59, 10.5)	-13.7 (-21.8, -5.64)^***^	-16.2 (-27.6, -4.78)	-1.08	0.01
IPAQ—SF						
SB/Sitting, min/day	6	-101 (-229, 26.3)	67.0 (-60.7, 194.8)	168.5 (-12.2, 349.2)	0.71	0.07
	12	-84.1 (-216, 47.6)	81.3 (-50.4, 213.0)	165.4 (-20.9, 351.6)	0.68	0.08
Walking (LPA), min/wk	6	-19.1 (-308, 269.6)	-113 (-402, 175.3)	-94.3 (-503, 314.0)	-0.18	0.64
	12	71.6 (-226, 369.2)	-191 (-488, 106.9)	-262 (-683, 158.6)	-0.47	0.22
MVPA, min/wk	6	-121 (-668, 424.9)	62.3 (-540, 664.3)	183.7 (-629, 996.6)	0.17	0.65
	12	149.0 (-452, 750.0)	-50.6 (-598, 497.1)	-200 (-1013, 613.5)	-0.19	0.62

^*^p value < 0.05; ^**^p value < 0.01; ^***^p value < 0.001

^*a*^Results are from unadjusted models, with similar results in adjusted models that included age and comorbidities as covariates

^*b*^Calculated as the mean/standard deviation of the difference between groups

^*c*^Sitting time only

^*d*^Sitting time + awake lying time

*BDS* = Borg Dyspnea Score; *CI* = confidence interval; *GAD-7* = Generalized Anxiety Disorder 7-items; *IPAQ-SF* = International Physical Activity Questionnaire – Short Form; *LPA* = light-intensity physical activity;* m6MWT* = mobile-based six-minute walk test; *MVPA* = moderate-vigorous intensity physical activity; *PSQI *= Pittsburgh Sleep Quality Index; *SB* = sedentary behavior; *SGRQ* = St. George’s Respiratory Questionnaire; *SES* = standardized effect size; *SOBQ* = University of California San Diego Shortness of Breath Questionnaire

## Discussion

In this pilot trial, we found that IMT + walking with behavioral support, compared to education only, was feasible, acceptable, safe, and could disrupt a “dyspnea-inactivity” spiral and improve HRQL among lung cancer survivors following curative intent therapy. These findings have important implications in efforts to reduce dyspnea and improve HRQL with this population.

**Fig. 2:**
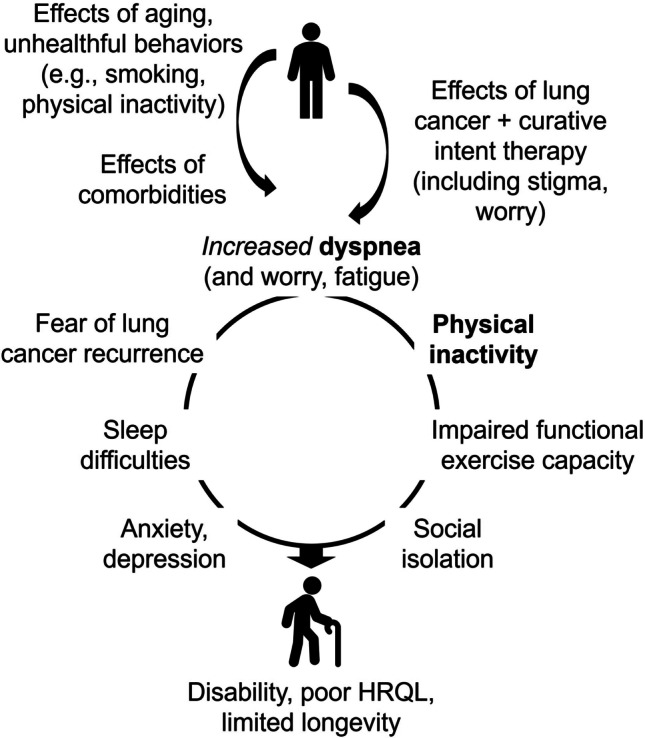
Conceptual Model of the Vicious Cycle of “Dyspnea-Inactivity” Downward Health Spiral Following Curative Intent Therapy of Lung Cancer. ***Bolded**** text indicates a vicious cycle of “dyspnea-inactivity.” Description *[4]*:* Following diagnosis and curative intent therapy of lung cancer, survivors experience increased symptom burden, particularly with dyspnea due to loss of lung tissue and function, lost or damaged nerve fibers and peripheral sensors, and alterations to the neuro-respiratory system, culminating in neuromechanical dissociation and increased central ‘corollary discharge’. Consequently, many survivors avoid physical activity and exercise [17, 18], leading to a vicious cycle of “dyspnea-inactivity.” Over time, combined with worry or fear of lung cancer recurrence [73], sleep disturbance [71], fatigue [71], the adopted physical inactivity leads to deconditioning [74], impaired functional exercise capacity [75], social isolation [76], anxiety and depressive symptoms, resulting in physical and psychosocial disability [77]. This downward health spiral can go unrecognized and negatively impact HRQL. Interventions should **promptly** disrupt this downward health spiral and reduce symptom burden, increase physical activity, social engagement, and promote behavior change to improve HRQL and other outcomes. HRQL = health-related quality of life

The US National Academy of Medicine recommends that care for posttreatment cancer survivors include supportive services to reduce treatment adverse effects and promote health [59]. Exercise is recommended by national [60, 61] and international societies [62] for cancer survivors. However, evidence on exercise benefits is mostly derived from survivors of breast, prostate, and colorectal cancer [62], with inconsistent evidence in lung cancer [63]. A systematic review involving lung cancer survivors within 12 months of surgical treatment demonstrated benefits of aerobic and resistance training on functional exercise capacity but low-to-very-low certainty evidence on dyspnea and HRQL [64]. We propose that exercise training and rehabilitative strategies to improve HRQL for lung cancer survivors following curative intent therapy may need to consider unique characteristics, including dyspnea, high cardiopulmonary disease burden, older age, pathophysiological/biobehavioral mechanisms, and promptly target specific impairments to disrupt a downward health spiral (Fig. 2).

Our trial supports the vicious cycle of “dyspnea-inactivity” conceptual model and the promise of IMT + walking as a targeted rehabilitative strategy for lung cancer survivors following curative intent therapy. In a small sample, we found that IMT + walking at 1–6 months posttreatment could reduce symptom burden, mitigate the negative impact of symptoms on social functioning and psychological disturbances, and improve HRQL. These potential benefits appeared to be sustained, possibly due to an behavioral support component informed by strong evidence derived from general adult population [35], with supporting evidence among cancer survivors [37, 38], including in a 2023 systematic review [65], and persisted in adjustments for age and comorbidities – two important characteristics in lung cancer. These potential benefits should be confirmed in larger trials with longer follow-up. IMT + walking had no statistically significant benefit on dyspnea, functional exercise capacity, anxiety, or sleep difficulties, possibly due to the small sample size and/or lack of additional targeted strategies (e.g., extremity strength training to increase functional exercise capacity), or in-person supervised exercise sessions. ActivPAL MVPA and SB improved at week 6 but waned at week 12, suggesting that reduced symptom burden could have mediated improved HRQL.

Feasibility was high regarding participant adherence to tele-visits, unsupervised IMT + walking, completion of activity logs and PCOMs, and retention. Enrollment was challenging, although comparable to other exercise trials in cancer [38] and higher than US National Clinical Trials in lung cancer [66], traditionally difficult to enroll [67]. Decreased intervention intensity or additional components, with compelling outcome data aligned with patient values or goals, may be needed for wider uptake. Acceptability to telemedicine-based IMT + walking was also high, with safety complementing a systematic review of in-person exercise training among post-surgical lung cancer survivors [64].

### Study strengths, limitations, and future directions

Strengths include: 1) a contemporary sample of lung cancer survivors within 1–6 months following curative intent therapy; 2) evaluation of a novel targeted intervention to disrupt a vicious cycle of “dyspnea-inactivity”; 3) block randomization to ensure balance between groups in a heterogenous population; 4) high feasibility and acceptability; 5) monitoring of safety events that included emergency room visits/hospitalizations in both groups; and 6) telemedicine-based delivery with minimal equipment and interventionist training, enhancing scalability and geographic reach potential.

Study limitations include participant adherence to IMT assessed by self-report, predisposing to social desirability and reporting bias. However, adherence measures completion was high, with step counts obtained by participants from Fitbit devices. Second, the m6MWT has not been evaluated in lung cancer, with responsiveness of the m6MWT distance is not known with certainty, precluding conclusions on feasibility. Nevertheless, functional exercise capacity is associated with HRQL [68], curative intent therapy outcomes, survival [69], is widely-used in exercise/rehabilitation trials [64], and has in-lab MCIDs available in lung cancer [70]. Third, we did not blind the outcome assessor nor use a sham intervention; however, we measured physical activity with the well-validated/highly-accurate activPAL. Fourth, while the vicious cycle of “dyspnea-inactivity” may be an important concept, we did not include other exercise training components, psychosocial interventions, nor strategies to improve sleep/fatigue [71]. Notwithstanding, IMT + walking could be a targeted strategy for dyspneic and physically inactive patients. Fifth, we lack a validated composite HRQL measure for disease-free lung cancer survivors. However, SGRQ subdomains align with the World Health Organization International Classification on Functioning, Disability and Health – a biopsychosocial model that incorporates biological, individual, and social perspectives on health and disability [72]. HRQL measures that include symptoms and treatment effects of advanced/metastatic lung cancer have limited utility for our target population. Sixth, our trial was designed to detect a very-large effect size difference in activPAL steps/day and thus was likely inadequately powered to detect changes in other PCOMs, precluding conclusion on treatment effects. Nevertheless, pilot trials are foundational to efficacy/effectiveness trials [42, 43]. Finally, all participants were from one healthcare system, most of white race/ethnicity, with lower-than-expected COPD prevalence, limiting generalizability.

Future trials can consider baseline dyspnea and physical activity levels to reduce participant heterogeneity, investigation of biophysiological mechanisms relating dyspnea and IMT, incorporate additional components, possibly with novel randomized trial designs (e.g., multiphase optimization strategy, hybrid, adaptive, platform) to encourage uptake, completion, and clinical translation.

## Conclusion

We conclude that telemedicine-based IMT + walking is feasible, acceptable, safe, and could disrupt the vicious cycle of “dyspnea-inactivity” downward health spiral among lung cancer survivors following curative intent therapy. These results warrant further investigations, including in prospective observational studies with longer follow-up, and larger, adequately powered randomized clinical trials.

## Supplementary information

Below is the link to the electronic supplementary material.

Supplementary file1 (DOCX 464 KB)

## Abbreviations

BDS: Borg Dyspnea Score
BMI: Body-mass index
CI: Confidence interval
COPD: Chronic obstructive pulmonary disease
ED: Emergency department
FEV_1_: Forced expiratory volume in 1 s
FVC: Forced vital capacity
GAD-7: Generalized Anxiety Disorder 7-item
HRQL: Health-related quality of life
IMT: Inspiratory muscle training
IPAQ-SF: International Physical Activity Questionnaire – Short Form
KPCO: Kaiser Permanente Colorado
LPA: Light intensity physical activity
m6MWT: Mobile-based six-minute walk test
MCID: Minimal clinically important difference
MVPA: Moderate-vigorous intensity physical activity
NSCLC-NOS: Non-small cell lung cancer, not otherwise specified
PA: Physical activity
PAGAC: Physical Activity Guidelines Advisory Committee
PCOM: Patient-centered outcome measure
PSQI: Pittsburgh Sleep Quality Index
RS: Research specialist
SB: Sedentary behavior
SBRT: Stereotactic body radiotherapy
SD: Standard deviation
SCT: Social Cognitive Theory
SES: Standardized effect size
SGRQ: St. George’s Respiratory Questionnaire
SOBQ: University of California Shortness of Breath Questionnaire
