# Supplementary material for: Telemedicine-based inspiratory muscle training and walking promotion with lung cancer survivors following curative intent therapy: a parallel-group pilot randomized trial
Source: Support Care Cancer. 2023 Sep 1;31(9):546. doi: 10.1007/s00520-023-07999-7 (PMC10474183; doi:10.1007/s00520-023-07999-7)
Supplement: Supplementary file 1 [file 520_2023_7999_MOESM1_ESM.docx]

**SUPPLEMENTARY MATERIALS**

Telemedicine-Based Inspiratory Muscle Training and Walking Promotion with Lung Cancer Survivors Following Curative Intent Therapy: A Parallel-Group Pilot Randomized Trial

*Supportive Care in Cancer*

**Online Resource 1:** Electronic Algorithm to Identify Patients for Recruitment

International Statistical Classification of Diseases (ICD) codes for lung cancer: C34, C7A.090, D38.1, D38.5, D38.6, D02.20, D02.22, D02.3, D02.4; suspected lung cancer: R91.1, R91.8; and Current Procedural Terminology (CPT) codes for thoracotomy or thoracoscopic lung resection surgery: 32096, 32097, 32100, 32110, 32120, 32440, 32442, 32445, 32480, 32482, 32484, 32486, 32488, 32491, 32501, 32503-32507, 32553, 32607, 32608, 32663, 32666-32671, 39000, 39010, 247135), radiation (+77293, 77373, 77385-77386, 77387, 77402, 77407, 77412, 77427, 77431, 77435, G6001, G6002, G6003-G6014, G6015-G6016, G6017 ), and radiation treatment planning (77261-77263 ). 

	While we did not conduct a formal evaluation of the sensitivity, specificity, and yield of each recruitment approach [i.e., 1) identification of patients with receipt of curative intent therapy; 2) new referrals to the pulmonology, surgery, or radiation oncology departments for newly-diagnosed or suspected lung cancer; and 3) patients presented at a weekly lung cancer/nodule conference], we found that approach # 1) (i.e., identification of patients with receipt of curative intent therapy) had the highest yield, approximately 20%. However, an important consideration is that this approach relies on accurate coding to capture procedures, particularly CPT codes for surgery and radiation treatment, and therefore may have limited generalizability to healthcare systems that do not no reliably code these procedures (i.e., particularly in settings in which physicians/surgeons do not need to code for the purpose of work capture or billing for financial reimbursement) or have an integrated electronic medical records system with accessible data.

**Online Resource 2:** Checklists of Intervention Components 

(A) **Initial Tele-visit**

| Intervention Checklist – Session 1 Participant #:                                       Session #:                                       Date: |  |  |
| --- | --- | --- |
| Component | Description | Y/N |
| Orientation | RS introduces program components (education, IMT + walking, and behavioral support) RS goes over educational materials on importance of exercise following lung cancer |  |
|  | RS inquires about baseline shortness of breath and walking behavior |  |
| Introduction to IMT | RS introduces IMT and explains how IMT can alleviate or improve dyspnea symptom and promote walking |  |
|  | RS demonstrates IMT technique |  |
|  | RS asks participants to demonstrate IMT technique and assist with supportive correction if needed |  |
|  | RS asks participant to adjust resistance to “somewhat hard” to hard” level |  |
|  | RS asks participant to report Borg Rating of Perceived Exertion (0-10 scale) and adjust as needed, to level of 4-6 (somewhat-hard to hard) |  |
| Introduction to Fitbit and walking program | RS explains the importance of regular walking |  |
|  | RS reviews Fitbit step count and walking behavior in previous week RS discusses importance syncing Fitbit data |  |
|  | RS troubleshoots with participant any difficulty with Fitbit wear, sync, or account access |  |
|  | RS shares average step count, discuss walking bouts identified, and supportively advises 5-10% increase |  |
| Health & safety | RS asks about 1) hospitalizations or emergency room (ER) visits; 2) illness or health problems where participants did not see a doctor; and 3) any other health issues in the past week |  |
|  | RS asks about falls or any other adverse events with walking or other exercises in the past week |  |
| Goal setting | RS explains/provides IMT + walking prescription and collaboratively establish goals for the upcoming 2 weeks |  |
|  | RS describes idea of creating an action plan with daily to weekly IMT + walking goals (e.g., alternating days of IMT and walking during weekday, then combined IMT + walking during weekend |  |
| Self-monitoring | RS explains use of Fitbit to facilitate monitoring of walking goals |  |
| Barriers/facilitators | RS + participant collaboratively discuss potential problems to achieving IMT + walking goals |  |
| Problem solving | RS + participant collaboratively identify strategies to minimize barriers and/or promote facilitators towards IMT + walking goal attainment |  |
| Encouragement | RS + participant review action plan for upcoming 2 weeks |  |
|  | RS encourages participant to engage in regular IMT + walking |  |

(B) **Subsequent Tele-visits** 

| Intervention checklist – Subsequent Sessions (weeks 3, 5, 7, 9, 12) Participant #:                                Session #:                                       Date: |  |  |
| --- | --- | --- |
| Component | Description | Y/N |
| Review program goals & educational materials | RS reviews program goals & educational materials on exercise following lung cancer treatment |  |
| IMT Demonstration & Training | RS asks participant to demonstrate IMT technique and re-train as needed |  |
|  | RS assesses IMT Rating of Perceived Exertion (Borg 0-10) & ask participant to re-adjust resistance level if needed (to 4-6, somewhat-hard to hard) |  |
|  | RS engages participant in 15 min of IMT (optional) |  |
| Review activity log | RS reviews health & safety adverse events in past 2 weeks (since last visit) |  |
|  | RS reviews the total number of IMT sessions in past 2 weeks |  |
| Review Fitbit data | RS reviews average Fitbit step count and walking bouts in past 2 weeks with participant |  |
| Barriers/facilitators | RS + participant collaboratively discuss problems to achieving IMT + walking goals, if any |  |
| Problem solving | RS + participant collaboratively identify strategies to minimize barriers and/or promote facilitators towards IMT + walking goal attainment, if any |  |
| Self-monitoring | RS emphasizes use of Fitbit to facilitate monitoring of walking goals |  |
| Goal setting | RS provides IMT + walking prescription and collaboratively establish goals for upcoming 2 weeks |  |
| Encouragement | RS + participant discuss review action plan for upcoming 2 weeks |  |
|  | RS encourages participant to engage in regular IMT + walking |  |

**Online Resource 3:** Mobile-Based Six-Minute Walk Test (m6MWT)

MOBILE-BASED SIX-MINUTE WALK TEST 

*(modified from the American Thoracic Society/ recommendations* *[17]* *with reference equations* *[18]**)*

Age:____	

Sex: M___   F___		

Race: White___; Hispanic___; Black___; Asian___; Other___	

Weight:____lbs, or____kg; 		

Height:____ft____in, or ____meters	

*Patient instructions:* 

*(to identify walk path):*

“To perform the 6-minute walk test, we ask for you to identify a walk path that is approximately 30 meters (100 feet) in length to walk back and forth; this is typically 1/3rd to 1/4th of a block in an urban city. Examples of walk paths include sidewalks, empty park space, large workout areas/gyms, parking lots, or hallways. If the path is not available or if weather does not permit, 15-meter (50-feet) walk paths, such as in a large living room in a US home, can be used instead. Because back-and-forth movements are important for this test, we ask that you NOT use circular or continuous walk paths. Also, the path should be free of obstables (avoiding busy spaces) and on level ground (and not on an incline).

“Please tell us the walk path you will use:

___Approximately 30 meters (or 100 feet) (e.g., sidewalk, park space, large workout area/gym, parking lot, or hallway)”

___Approximately 15 meters (or 50 feet) (e.g., living room, basement, small workout area/gym)  

Supplemental oxygen during the test: No___	Yes___, flow_____L/min 

Facial mask/covering during the test: No___		Yes___

*(to assess resting and exertional dyspnea levels)*

 “Please rate your current level of shortness of breath between 0 and 10, where 0 is nothing at all, and 10 is very, very severe (or maximal) shortness of breath (Borg Dyspnea Scale).

*Pre-6MWT (resting) Borg Dyspnea Score (0-10 scale):____*

*(for performance, read to patient):* 

“The objective of this test is to walk as far as possible for 6 minutes. You will walk back and forth on the walk path that you selected. Six minutes is a long time to walk, so you will be exerting yourself. You will probably get out of breath or become exhausted. Your are permitted to slow down, to stop, and to rest as necessary, but resume walking as soon as you are able. If you stop and rest, please allow the mobile 6-minute walk application to continue recording time. 

“As you walk back and forth, each time you get to the end of the walk path, you should turn around quickly and walk back the other way without hesitation. This is so that you will achieve the longest possible distance without losing time.

“Are you ready to do that? When you are ready, please press START on the mobile 6-minute walk application and start walking.”

*After 6 minutes, record End of Test Borg Dyspnea Score*: “Please rate your current level of shortness of breath between 0 and 10, where 0 is nothing at all, and 10 is very, very severe (or maximal) shortness of breath (Borg Dyspnea Scale).”

End of 6MWT Borg Dyspnea Score (0-10 scale):____

*Total distance walked in 6 minutes (6MWD)*:_______feet, or _______meters  

*Stopped or paused before 6 minutes?* No____	Yes___, reason:________________

*Predicted* 6MWD:______meters; 		Percent predicted:_______%

**Online Resource 4:** Description of Patient-Centered Outcome Measures

The University of California San Diego Shortness of Breath Questionnaire (SOBQ) is a 24-item measure of dyspnea with a variety of activities of daily living in patients with chronic lung disease [1]. Reponses are recorded on Likert scales ranging 0-5, summed (total scores range 0-120), with higher scores indicating higher levels of dyspnea. Significant dyspnea levels have been described as SOBQ > 9 points in patients with COPD [2]. The minimal clinically important difference (MCID) is ≥ 5 points [3]. The SOBQ has been used in lung cancer survivors following curative intent therapy [4].

The Generalized Anxiety Disorder 7-item (GAD-7) is a 7-item measure that uses some of the Diagnostic and Statistical Manual of Mental Disorder criteria for general anxiety disorder to assess anxiety symptom severity in the past two weeks [5]. Responses are recorded on a Likert scale ranging 0-3, summed, with total scores categorized to indicate minimal (0-4), mild (5-9), moderate (10-14), and severe (15-21) anxiety symptoms. The MCID is ≥ 3 points [6].

The Pittsburgh Sleep Quality Index (PSQI) is a 9-item measure of sleep quality, sleep latency, sleep duration, habitual sleep efficiency, sleep disturbance, use of sleep aids, and daytime dysfunction in the past one month [7]. Responses are recorded on 0-3 Likert scales, summed (total scores range 0-21), with poor/disrupted sleep defined as total scores ≥ 5. A MCID threshold of 3 points has been suggested [8].

The St. George’s Respiratory Questionnaire (SGRQ) is a 50-item measure of quality of life for patients with chronic obstructive pulmonary disease (COPD) [9] that has also been used to assess the health-related quality of life (HRQL) of early stage lung cancer survivors [10]. The SGRQ assesses three components of HRQL: symptoms (frequency and severity), activities that cause or are limited by dyspnea, and impact (of chronic airway disease on social functioning, psychological disturbance). Response items are weighted to generate total scores that range 0-100, with higher scores indicating worse HRQL. Thresholds of impaired HRQL has been suggested as SGRQ > 13 points in patients with COPD [11]. The MCID is 4 SGRQ points [12]. 

The International Physical Activity Questionnaire – Short Form (IPAQ-SF) is a 7-item measure of vigorous and moderate intensity physical activity, walking, and sedentary (sitting) behaviors in the past seven days [13]. Reponses are recorded as integers and multiplied to generate minutes/week or minutes/day of the respective behaviors. Scores can be categorized or summarized as a continuous measure, calculated using multipliers of metabolic equivalent of tasks (METs) and expressed as MET-minute/week [14]. For simplicity and ease of interpretation, as our intervention encourages walking, we used minutes/week of the respective physical activity (walking or moderate-vigorous intensity) behavior for summary statistics and analysis. This approach has been used in another study involving cancer survivors [15]. A MCID threshold of 13 minutes/week (of moderate-to-vigorous activity) has been suggested [16]. The MCID for sedentary behavior is not available. 

**Online Resource 5:** Participant Adherence^***^ to IMT + Walking

a) Self-report of IMT each week

b) Self-report of walking each week

^***^*Obtained from patient-reported activity logs, with 11 (100%) completed logs/week in weeks one to eight, 10 (91%) completed logs in week nine, 8 (73%) completed logs/week in weeks ten and eleven, and 7 (64%) completed logs in week twelve.*

**Online Resource 6:** Change in Patient-Centered Outcome Measures from Baseline to Follow Up^***^

 

^***^*At six and/or 12 week follow up.*

^*a*^*Higher MVPA indicates improved levels of physical activity.* 

^*b*^*Lower SB indicates improved sedentary behavior (SB-2 includes awake lying time).*

^*c*^*Lower scores (0-100 point scale) indicate improved HRQL.*

*Dashed grey lines represent individual participants in both intervention and control groups.*

*Red (or black on grey scale) solid line represents mean values of participants in the intervention group.*

*Grey solid line represents mean values of participants in the control group.*

HRQL = health-related quality of life; MPVA = moderate-vigorous intensity physical activity; SB = sedentary behavior; SGRQ = St. George’s Respiratory Questionnaire

**References**

1.	Eakin EG, Resnikoff PM, Prewitt LM, et al. Validation of a new dyspnea measure: the UCSD Shortness of Breath Questionnaire. University of California, San Diego. Chest. 1998;113(3):619-24.

2.	Martinez FJ, Foster G, Curtis JL, et al. Predictors of mortality in patients with emphysema and severe airflow obstruction. American journal of respiratory and critical care medicine. 2006;173(12):1326-34.

3.	Kupferberg DH, Kaplan RM, Slymen DJ, et al. Minimal clinically important difference for the UCSD Shortness of Breath Questionnaire. Journal of cardiopulmonary rehabilitation. 2005;25(6):370-7.

4.	Ha D, Ries AL, Mazzone PJ, et al. Exercise capacity and cancer-specific quality of life following curative intent treatment of stage I-IIIA lung cancer. Support Care Cancer. 2018;26(7):2459-69.

5.	Spitzer RL, Kroenke K, Williams JB, et al. A brief measure for assessing generalized anxiety disorder: the GAD-7. Archives of Internal Medicine. 2006;166(10):1092-7.

6.	Kroenke K, Baye F, Lourens SG. Comparative Responsiveness and Minimally Important Difference of Common Anxiety Measures. Medical care. 2019;57(11):890-7.

7.	Buysse DJ, Reynolds CF, 3rd, Monk TH, et al. The Pittsburgh Sleep Quality Index: a new instrument for psychiatric practice and research. Psychiatry research. 1989;28(2):193-213.

8.	Hughes CM, McCullough CA, Bradbury I, et al. Acupuncture and reflexology for insomnia: a feasibility study. Acupuncture in medicine : journal of the British Medical Acupuncture Society. 2009;27(4):163-8.

9.	Jones PW, Quirk FH, Baveystock CM. The St George's Respiratory Questionnaire. Respiratory medicine. 1991;85 Suppl B:25-31; discussion 3-7.

10.	Nugent SM, Golden SE, Hooker ER, et al. Longitudinal Health-related Quality of Life among Individuals Considering Treatment for Stage I Non-Small-Cell Lung Cancer. Annals of the American Thoracic Society. 2020;17(8):988-97.

11.	Weatherall M, Marsh S, Shirtcliffe P, et al. Quality of life measured by the St George's Respiratory Questionnaire and spirometry. Eur Respir J. 2009;33(5):1025-30.

12.	Jones PW. St. George's Respiratory Questionnaire: MCID. Copd. 2005;2(1):75-9.

13.	Lee PH, Macfarlane DJ, Lam TH, et al. Validity of the International Physical Activity Questionnaire Short Form (IPAQ-SF): a systematic review. The international journal of behavioral nutrition and physical activity. 2011;8:115-5868-8-115.

14.	Guidelines for Data Processing and Analysis of the International Physical Activity Questionnaire (IPAQ) - Short Form 2004 April.

15.	Ruiz-Casado A, Alejo LB, Santos-Lozano A, et al. Validity of the Physical Activity Questionnaires IPAQ-SF and GPAQ for Cancer Survivors: Insights from a Spanish Cohort. Int J Sports Med. 2016;37(12):979-85.

16.	Hur SA, Guler SA, Khalil N, et al. Minimal Important Difference for Physical Activity and Validity of the International Physical Activity Questionnaire in Interstitial Lung Disease. Annals of the American Thoracic Society. 2019;16(1):107-15.

17.	Laboratories ATSCoPSfCPF. ATS statement: guidelines for the six-minute walk test. American journal of respiratory and critical care medicine. 2002;166(1):111-7.

18.	Enright PL, Sherrill DL. Reference equations for the six-minute walk in healthy adults. American journal of respiratory and critical care medicine. 1998;158(5 Pt 1):1384-7.

19.	Ha D, Ries AL, Lippman SM, et al. Effects of curative-intent lung cancer therapy on functional exercise capacity and patient-reported outcomes. Supportive care in cancer : official journal of the Multinational Association of Supportive Care in Cancer. 2020;28(10):4707-20.

20.	Parshall MB, Schwartzstein RM, Adams L, et al. An official American Thoracic Society statement: update on the mechanisms, assessment, and management of dyspnea. American journal of respiratory and critical care medicine. 2012;185(4):435-52.

21.	Granger CL, Connolly B, Denehy L, et al. Understanding factors influencing physical activity and exercise in lung cancer: a systematic review. Supportive care in cancer : official journal of the Multinational Association of Supportive Care in Cancer. 2017;25(3):983-99.

22.	Zheng W, Hu M, Liu Y. Social support can alleviate the fear of cancer recurrence in postoperative patients with lung carcinoma. Am J Transl Res. 2022;14(7):4804-11.

23.	Granger CL, Parry SM, Edbrooke L, et al. Deterioration in physical activity and function differs according to treatment type in non-small cell lung cancer - future directions for physiotherapy management. Physiotherapy. 2016;102(3):256-63.

24.	Granger CL, McDonald CF, Irving L, et al. Low physical activity levels and functional decline in individuals with lung cancer. Lung cancer (Amsterdam, Netherlands). 2014;83(2):292-9.

25.	Zhang N, He X, Zhang H, et al. Influencing Factors of Physical Activity in Patients with Lung Cancer Surgery and Its Correlation with Exercise Self-Efficacy and Perceived Social Support. Evid Based Complement Alternat Med. 2022;2022:7572530.

26.	Bade BC, Faiz SA, Ha DM, et al. Cancer-related Fatigue in Lung Cancer: A Research Agenda: An Official American Thoracic Society Research Statement. Am J Respir Crit Care Med. 2023;207(5):e6-e28.

27.	Joshy G, Thandrayen J, Koczwara B, et al. Disability, psychological distress and quality of life in relation to cancer diagnosis and cancer type: population-based Australian study of 22,505 cancer survivors and 244,000 people without cancer. BMC medicine. 2020;18(1):372-020-01830-4.

28.	Kocarnik JM, Compton K, Dean FE, et al. Cancer Incidence, Mortality, Years of Life Lost, Years Lived With Disability, and Disability-Adjusted Life Years for 29 Cancer Groups From 2010 to 2019: A Systematic Analysis for the Global Burden of Disease Study 2019. JAMA Oncol. 2022;8(3):420-44.
